# Robotic modified Strong procedure for superior mesenteric artery syndrome

**DOI:** 10.1002/ccr3.7651

**Published:** 2023-07-17

**Authors:** Kareem Wasef, Aaron Hudnall, Carl R. Schmidt, J. Wallis Marsh, Brian A. Boone

**Affiliations:** ^1^ Department of Surgery West Virginia University Morgantown West Virginia USA; ^2^ Department of Microbiology, Immunology and Cell Biology Morgantown West Virginia USA

**Keywords:** duodenal obstruction, robotic Strong procedure, Strong procedure, superior mesenteric artery syndrome

## Abstract

**Key Clinical Message:**

The robotic modified Strong procedure is a safe and effective approach for surgical management of superior mesenteric artery syndrome in properly selected patients.

**Abstract:**

Superior mesenteric artery syndrome is a rare syndrome of small bowel obstruction resulting from vascular compression of the duodenum. Here we present our modification of a robotic Strong procedure for the surgical management of SMA syndrome. This procedure is a safe and effective approach for management in properly selected patients.

## Graphical abstract

Preoperative upper GI imaging demonstrating narrowing of the third portion of the duodenum with diminished transit of contrast (left). Preoperative CTA demonstrating a narrowed aortomesenteric angle of 14.11 degrees, consistent with the diagnosis of SMAS (right).

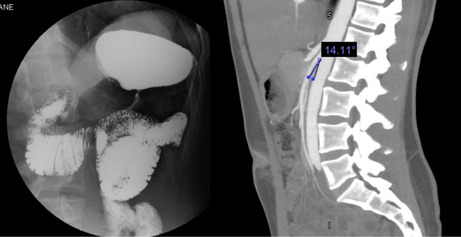

## 1 INTRODUCTION

Superior mesenteric artery syndrome (SMAS) is a condition resulting from vascular compression of the third part of the duodenum in the angle between the aorta and the superior mesenteric artery.^1^, ^2^ SMAS presents with symptoms caused by the duodenal obstruction including postprandial epigastric pain, nausea, vomiting, anorexia, and weight loss.^3^ This is a rare syndrome with reported prevalence estimated between 0.013% and 0.3%.^4^, ^5^ The etiology is related to factors diminishing the aortomesenteric angle and loss of mesenteric fat. Multiple risk factors have been identified including anatomic variants with high insertion of ligament of Treitz, severe weight loss, trauma, postoperative conditions, and scoliosis surgery.^1^ SMAS diagnosis can be established using upper gastrointestinal series documenting duodenal obstruction, cross sectional imaging with arterial phase contrast to show a narrowed SMA angle, and endoscopy to rule out other pathologies. These modalities classically demonstrate dilation of first and second part of duodenum, compression of mucosal folds, antiperistaltic flow of contrast medium, delay in transit through the gastroduodenal region, and relief of obstruction in a prone, knee‐chest or left lateral decubitus position.^3^, ^6^ Additionally, an aortomesenteric angle below 22 degrees and a distance less than 8 mm seen on contrast‐enhanced CT angiography is consistent with SMAS.^7^

Initial approaches to treatment of SMAS are conservative including gastric decompression, correction of fluid and electrolyte balance, enteral tube feeding or parenteral nutrition, and an attempt to increase the aortomesenteric angle by increasing body weight to restore retroperitoneal fat.^1^, ^8^, ^9^, ^10^ In the cases of failure of noninvasive therapy, surgical management is indicated to relieve the obstruction. Multiple surgical approaches have been described in the literature including gastrojejunostomy, duodenojejunostomy,^11^ infrarenal transposition of the superior mesenteric artery,^12^ and the Strong procedure.^13^ Strong procedure relies on division of the ligament of Treitz with mobilization of the duodenum for caudal displacement and offers the advantages of being less invasive, quicker, and safer with an earlier postoperative recovery.^14^, ^15^

Minimally invasive approaches using laparoscopic techniques have been adapted in the management of SMAS with both the Strong procedure^16^ and duodenojejunostomies.^17^, ^18^ More recently, robotic surgical approaches have also been described for duodenojejunostomies.^19^, ^20^ Robotic surgery offers numerous advantages including enhanced three‐dimensional visualization, instrument wrist articulation, and the ability for the operating surgeon to control multiple instruments and the camera. This results in reduced operative times and faster recovery for many procedures. To our knowledge, robotic Strong procedure has only been described once in the literature by Konstantinidis et al. in 2017.^21^ In this case report, we present our own technique for a modified robotic Strong procedure in the treatment of SMAS.

## 2 MATERIALS AND METHODS

Our patient was a 26‐year‐old female who had a several year history of nausea, vomiting, postprandial abdominal pain, and flushing, associated with an 80 pound weight loss. She had previously been managed with a PEG‐J tube and initially improved with gain of weight; however, her weight loss and symptoms recurred after the PEG‐J was removed. Her past medical history was significant for anxiety, depression and gastroesophageal reflux disease and her past surgical history was significant for colonoscopy and esophagogastroduodenoscopy.

**FIGURE 1:**
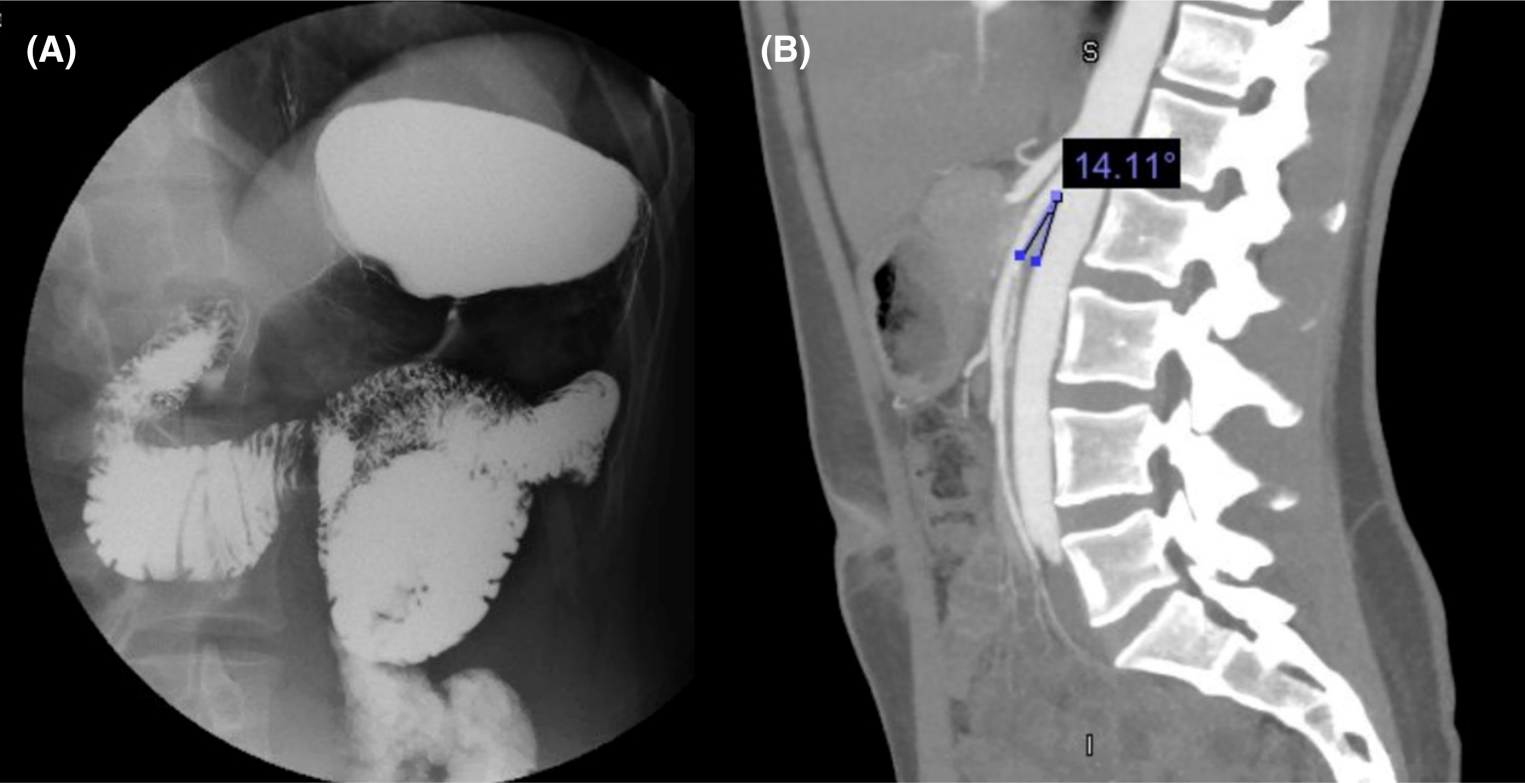
(A) Preoperative upper GI imaging demonstrating narrowing of the third portion of the duodenum with diminished transit of contrast (B) Preoperative CTA demonstrating a narrowed aortomesenteric angle of 14.11 degrees, consistent with the diagnosis of SMAS.

On upper GI imaging a focal narrowing of the 3rd portion of the duodenum with diminished transit of contrast was visualized (Figure 1A). During dynamic imaging, antiperistalsis against the narrowing was also demonstrated. CTA demonstrated an aorto‐SMA angle of approximately 14 degrees, more acute than the expected angle of 25–60 degrees and consistent with the diagnosis of superior mesenteric artery syndrome (Figure 1B). After discussing treatment approaches, a definitive surgical treatment using a robotic Strong procedure was decided upon.

## 3 RESULTS

### 3.1 Technical description (Video S1: technique for modified robotic Strong procedure)

**FIGURE 2:**
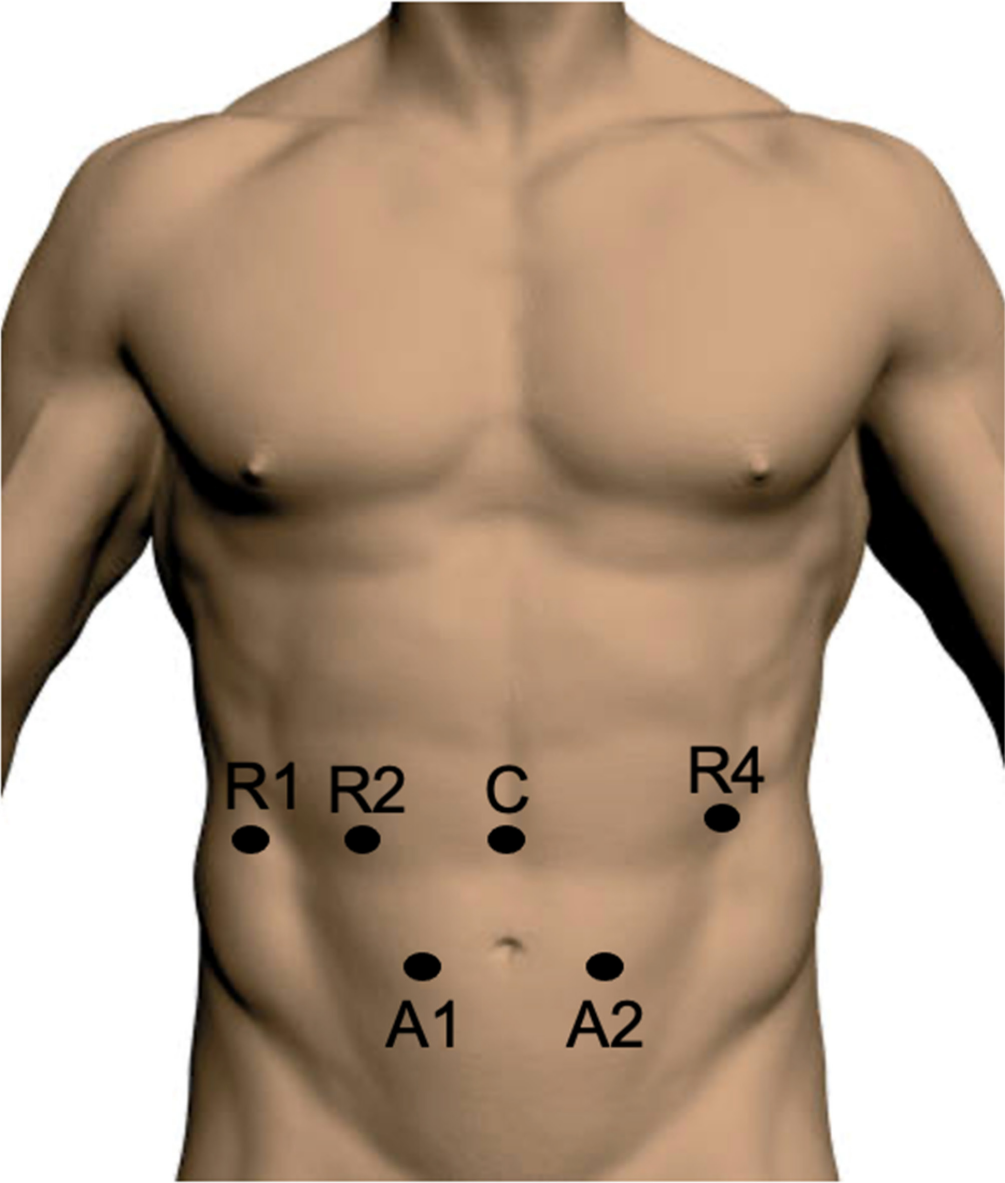
Port placement.

**FIGURE 3:**
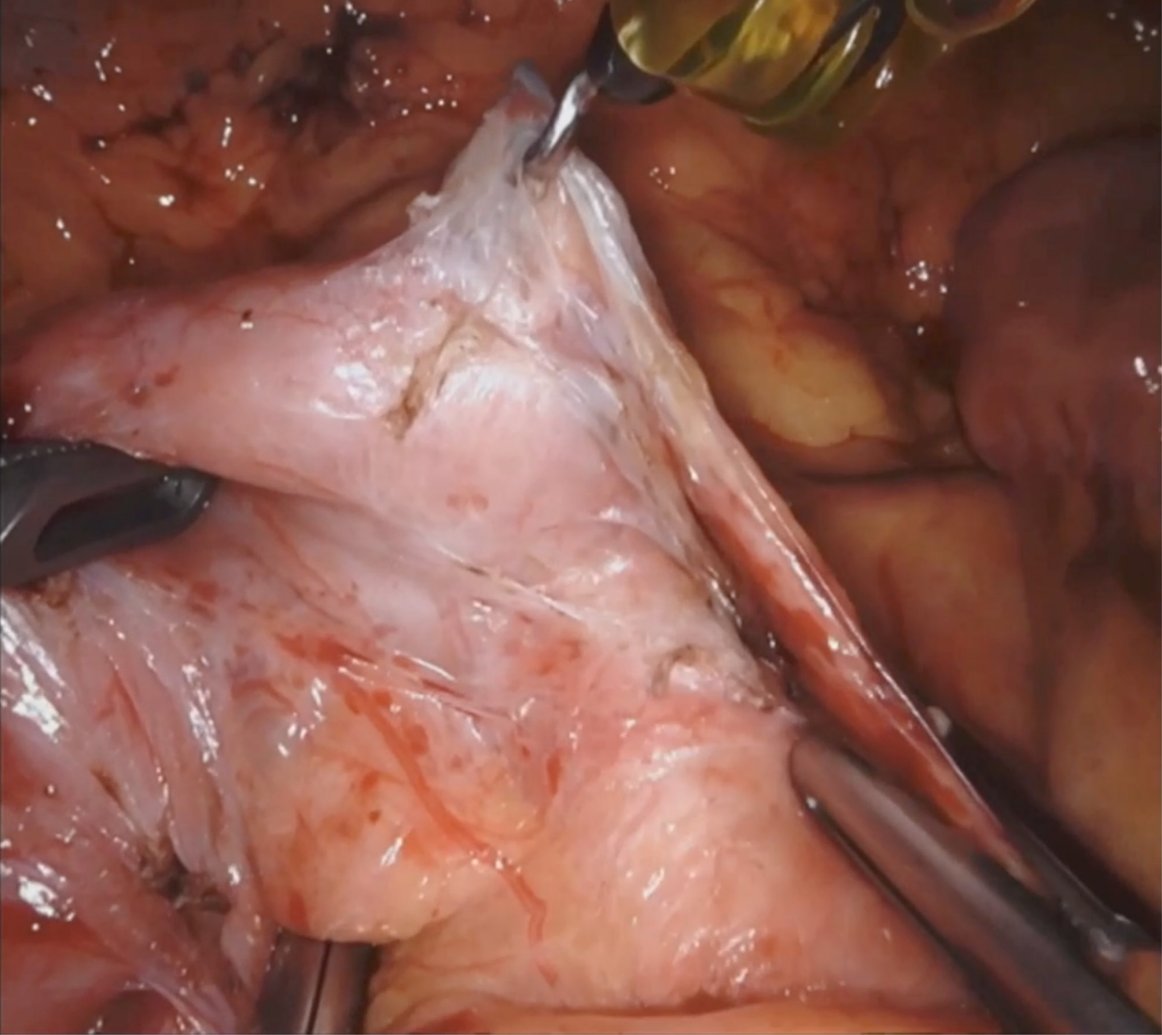
Intraoperative image showing use of Cadiere forceps for retraction in robotic arms 1/2 with hook electrocautery being used in robotic arm 4 and the assistant using atraumatic graspers to help with retraction and occasionally, a vessel sealing energy device.

The patient is placed supine on a split leg table. General anesthesia is induced, and endotracheal tube is passed. A left upper quadrant incision is made, and the optical separator device is used to gain access to the abdominal cavity. The abdomen is insufflated and explored. Four 8 mm robotic ports are placed over the upper abdomen and two 5 mm assistant ports in bilateral lower quadrants (Figure 2). The patient is then placed in reverse Trendelenburg and the robot is docked. The procedure begins by retracting the transverse colon cephalad and identifying the ligament of Treitz. The dissection begins by dissecting the ligament of Treitz from the left side. Throughout the procedure Cadiere forceps are used for retraction during the majority of the dissection in robotic arms 1 and 2, with hook electrocautery being used in robotic arm 4. Bipolar cautery is infrequently used in robotic arm 2. The assistant can use atraumatic graspers to help with retraction and occasionally, a vessel sealing energy device (Figure 3).

The peritoneum overlying the proximal jejunum at the ligament of Treitz is dissected using hook electrocautery. The bowel is retracted inferiorly to facilitate dissection. The inferior mesenteric vein is preserved and retracted laterally and cephalad to avoid injury during the dissection. The aorta is seen to the left of the proximal jejunum. This dissection is continued until all of the thin peritoneal attachments of the bowel are divided underneath the SMA.

The field of dissection then moves to the right of the ligament of Treitz. The anterolateral wall of the duodenum is identified, and a combination of electrocautery and blunt dissection is used to separate the duodenum from the transverse colon mesentery. The duodenum is then medialized, exposing the inferior vena cava. The plane of dissection during the Kocher maneuver close to the duodenum, but care must be taken to avoid injury. Once the 3rd portion of the duodenum is mobilized, the dissection proceeds cephalad along the anterior surface of the vena cava. This allows for further medial retraction of the duodenum to facilitate taking down additional retroperitoneal attachments at the ligament of Treitz. Eventually, this dissection joins the previous dissection, and a window is made posterior to the duodenum. The aorta is now visible from the right side of the ligament of Treitz.

Once the ligament is completely taken down, the proximal jejunum is delivered through the retroperitoneum using a robotic cadiere and the assistant bowel grasper. The dissection then proceeds back to the patients left side to further enlarge the RP window behind the SMA using electrocautery.

**FIGURE 4:**
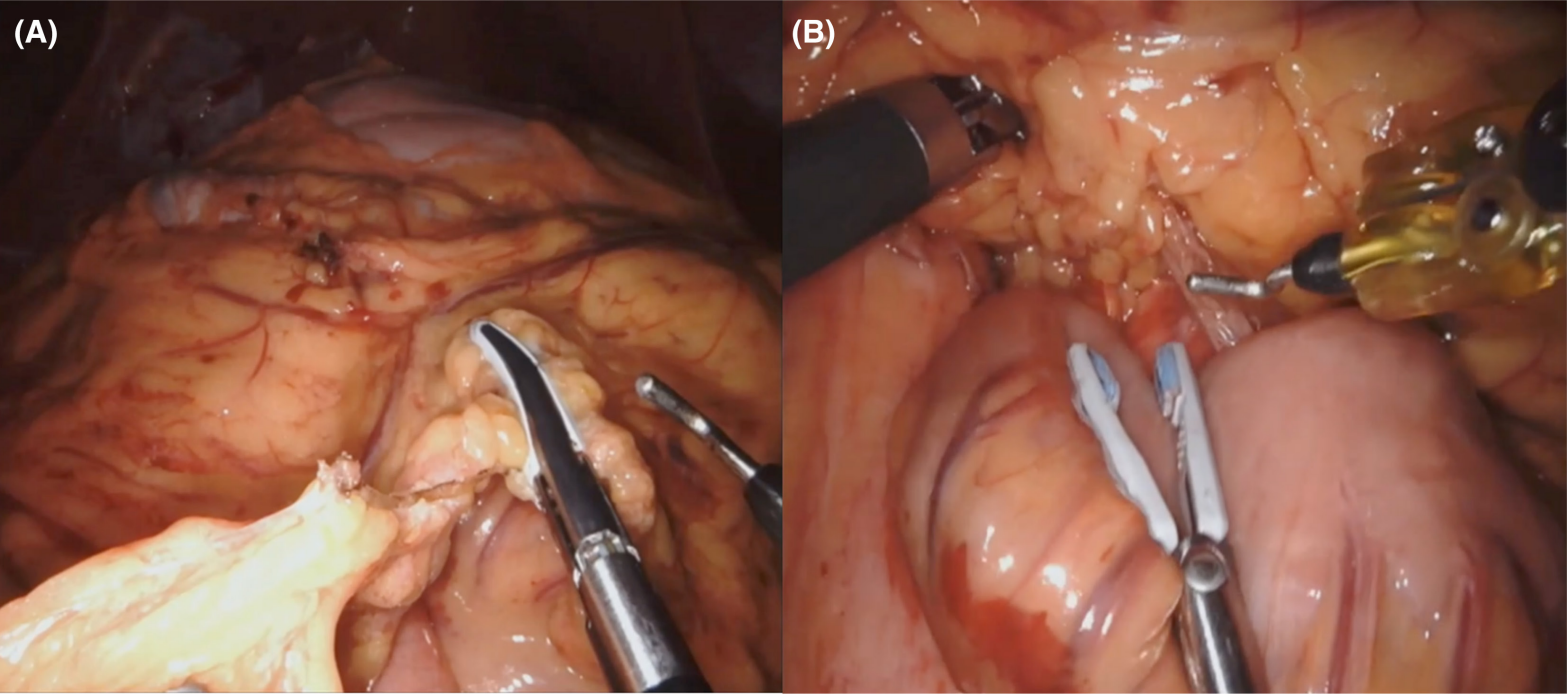
Intraoperative images (A) Mobilization of vascularized omental flap (B) Securing the omental flap at the apex of the angle between the SMA and aorta.

To prevent the bowel from slipping back into the acute angle between the SMA and aorta, a vascularized omental flap is mobilized. This is performed using the vessel sealing energy device to divide the omentum and then securing the omental flap into the space at the apex of the angle between the SMA and aorta (Figure 4). Additionally, the proximal jejunum can be sutured to the right upper quadrant abdominal wall to prevent slipping back through the retroperitoneal defect.

### 3.2 Perioperative outcome

**FIGURE 5:**
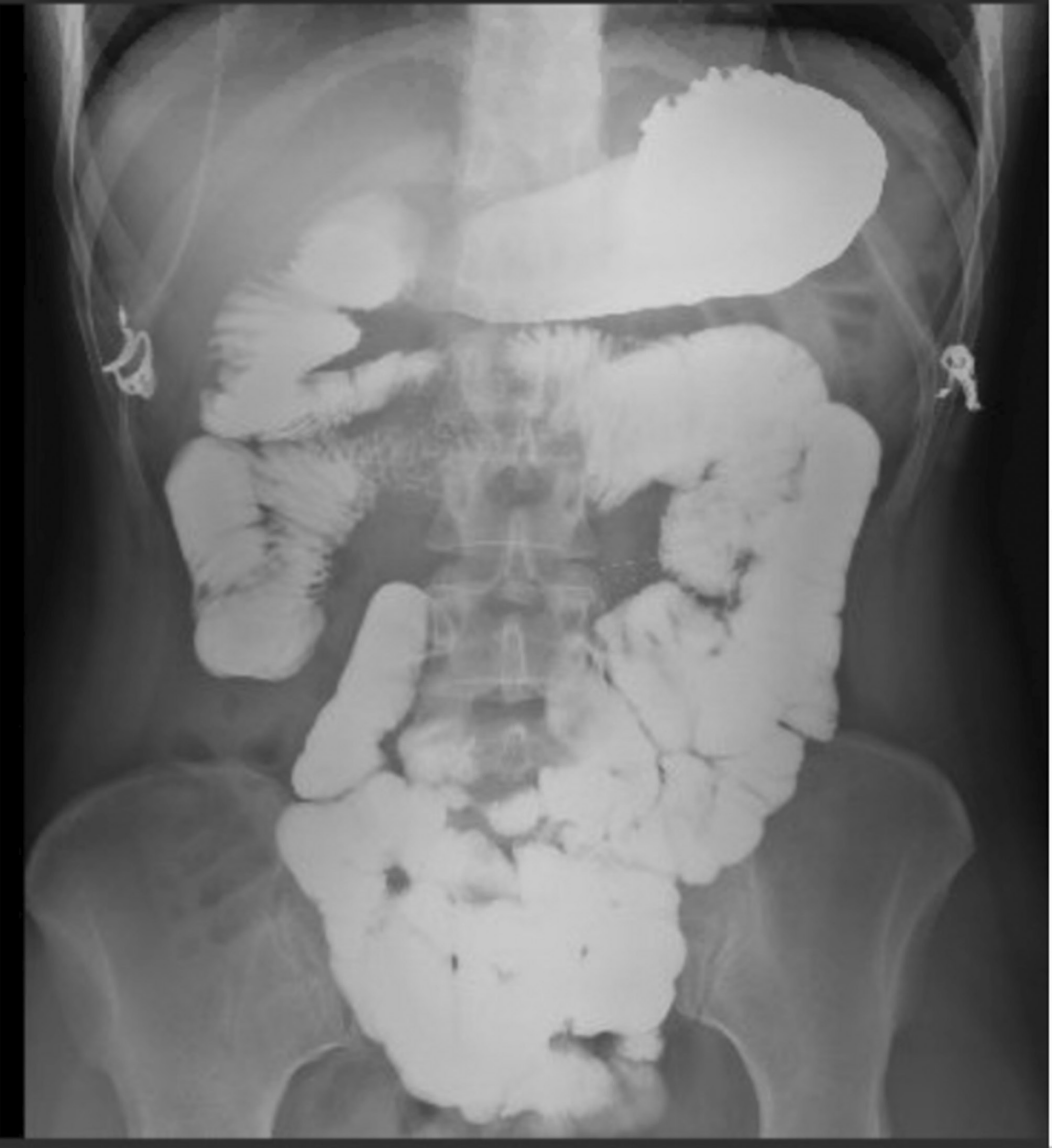
Postoperative upper GI imaging showing duodenum and mobilized jejunum in the right upper quadrant with good flow of contrast throughout the small bowel.

The operative time was 140 min. The recovery was favorable, postoperative GI imaging demonstrated resolution of the obstruction (Figure 5) and the patient was discharged on postoperative day 2 without complications. She was readmitted once with constipation and dehydration, however, dramatically improved with rehydration. She is currently eating very well with a good appetite and substantial weight gain over 2.5 years post‐op. She denies nausea, vomiting, or abdominal pain and no longer has postprandial flushing.

We have performed three of these procedures to date with an average operative time of 150.3 min +/− 9.5 min, and an average length of stay of 3 days, ranging from 2 to 5 days. There were no intraoperative complications and all patients had durable resolution of symptoms with increased oral intake and weight gain following the procedure.

## 4 DISCUSSION

SMAS is a rare condition that results from vascular compression of the third part of the duodenum in the angle between the aorta and the superior mesenteric artery.^1^, ^2^ This is thought to be related to factors causing diminishing of the aortomesenteric angle and loss of mesenteric fat. Some of the risk factors identified that contribute to this etiology include anatomic variants with high insertion of ligament of Treitz, severe weight loss, trauma, postoperative conditions, and scoliosis surgery.^1^ SMAS presents with symptoms caused by the duodenal obstruction including postprandial epigastric pain, nausea, vomiting, anorexia, and weight loss.^3^ The initial management is usually conservative and includes gastric decompression, correction of fluid and electrolyte balance, enteral tube feeding or parenteral nutrition, and an attempt to increase the aortomesenteric angle by increasing body weight to restore retroperitoneal fat.^1^, ^8^, ^9^, ^10^ When these measures fail, surgery is indicated, with many techniques described in the literature including gastrojejunostomy, duodenojejunostomy,^11^ infrarenal transposition of the superior mesenteric artery,^12^ and the Strong procedure.^13^

When compared to other popular surgical approaches for treating SMAS, such as duodenojejunostomy and gastrojejunostomy, the Strong procedure offers the advantages of being less invasive, quicker, and safer, with no risk of anastomotic leak.^14^, ^15^ Avoiding anastomoses in these malnourished patients is an important consideration and a limitation of other surgical approaches. Furthermore, gastrojejunostomy has been shown to provide adequate decompression of the stomach but may fail to release the duodenal obstruction leading to significant bile reflux and blind loop syndrome, requiring further surgical intervention.^11^ Duodenojejunostomy has been reported to have success rates around 80%,^1^, ^11^ but has the disadvantages of requiring anastomoses and altering the digestive tract physiology.^22^ Strong procedure has been reported to have success rates of up to 95%,^22^ while offering the advantages discussed above.

Additionally, our modification of the original Strong procedure involves the addition of the omental flap, which prevents the bowel from sliding cephalad and maintains a greater angle between the SMA and aorta. Performing this procedure using the robotic platform capitalizes on its technical advantages including enhanced visualization and instrument articulation, which has the potential to reduce operative times, speed recovery, and enhance cosmetic outcomes compared to an open approach.

Potential pitfalls involved with the described approach are uncommon, but include recurrence of symptoms, injury to the bowel during manipulation and bleeding from the inferior mesenteric vein during dissection at the ligament of Treitz. Because a surgical bypass is not performed, there is a possibility that the aortomesenteric angle narrows again or that the jejunum slips back to its normal anatomic location. We performed the omental flap to reduce this risk, and have not experienced this, but it remains possible. If symptoms reoccur after a Strong procedure, then a gastrojejunal or duodenojejunal bypass could be considered.

## 5 CONCLUSIONS

The robotic Strong procedure is a safe and effective approach for surgical management of SMAS and is associated with favorable clinical outcomes.

## AUTHOR CONTRIBUTIONS

**Kareem Wasef:** Data curation; writing – original draft; writing – review and editing. **Aaron Hudnall:** Software; writing – review and editing. **Carl Schmidt:** Methodology; writing – review and editing. **James Wallis Marsh:** Methodology; writing – review and editing. **Brian Boone:** Conceptualization; data curation; methodology; writing – review and editing.

## FUNDING INFORMATION

No funding was received for this study.

## CONFLICT OF INTEREST STATEMENT

The authors have no disclosures and no conflicts of interest to report.

## ETHICS STATEMENT

The study was approved by the West Virginia University Institutional Review Board (#2105312839). The authors have received and archived patient consent for video recording/publication in advance of video recording of procedure.

## CONSENT

Written informed consent was obtained from the patient to publish this report in accordance with the journal's patient consent policy.

## Supporting information


Video S1

## Data Availability

The data that support the findings of this study are available on request from the corresponding author. The data are not publicly available due to privacy or ethical restrictions.

## References

[1] Welsch T , Büchler MW , Kienle P . Recalling superior mesenteric artery syndrome. Dig Surg. 2007;24:149‐156. doi:10.1159/000102097 17476104

[2] Barchi L , Alves A , Jacob C . Favorable minimal invasive surgery in the treatment of superior mesenteric artery syndrome: case report. Int J Surg Case Rep. 2016;29:223‐226. 27914348 10.1016/j.ijscr.2016.09.016 PMC5133654

[3] Dietz U , Debus E , Heuko‐Valiati L , et al. Aorto‐mesenteric artery compression syndrome. Chirurg. 2000;71(11):1345‐1351. 11132320 10.1007/s001040051224

[4] Goin L , Wilk S . Intermittent arteriomesenteric occlusion of the duodenum. Radiology. 1956;67:729‐737. 13370885 10.1148/67.5.729

[5] Nugent F , Braasch J , Epstein H . Diagnosis and surgical treatment of arteriomesenteric obstruction of the duodenum. JAMA. 1966;196:1091‐1093. 5952480

[6] Hines J , Gore R , Ballantyne G . Superior mesenteric artery syndrome. Diagnostic criteria and therapeutic approaches. Am J Surg. 1984;148:630‐632. 6496852 10.1016/0002-9610(84)90339-8

[7] Unal B , Aktas A , Kemal G , et al. Superior mesenteric artery syndrome: CT and ultrasonography findings. Diagn Interv Radiol. 2005;11:90‐95. 15957095

[8] Lippl F , Hannig C , Weiss W , et al. Superior mesenteric artery syndrome diagnosis and treatment from the gastroenterologist's view. J Gastroenterol. 2002;37:640‐643. 12203080 10.1007/s005350200101

[9] Shin M , Kim J . Optimal duration of medical treatment in superior mesenteric artery syndrome in children. J Korean Med Sci. 2013;28:1220‐1225. 23960451 10.3346/jkms.2013.28.8.1220 PMC3744712

[10] Shiu J , Chao H , Luo C . Clinical and nutritional outcomes in children with idiopathic superior mesenteric artery syndrome. J Pediatr Gastroenterol Nutr. 2010;51:177‐182. 20601910 10.1097/MPG.0b013e3181c7bdda

[11] Lee C , Mangla J . Superior mesenteric artery compression syndrome. Am J Gastroenterol. 1978;70:141‐150. 717365

[12] Pourhassan S , Grotemeyer D , Furst G , et al. Infrarenal transposition of the superior mesenteric artery: a new approach in the surgical therapy of Wilkie syndrome. J Vasc Surg. 2008;47:201‐204. 17949939 10.1016/j.jvs.2007.07.037

[13] Strong E . Mechanics of arteriomesenteric duodenal obstruction and direct surgical attack upon etiology. Ann Surg. 1958;148:725‐730. 13595530 10.1097/00000658-195811000-00001 PMC1450911

[14] Burrington J . Superior mesenteric artery syndrome in children. Am J Dis Child. 1976;130:1367‐1370. 998581 10.1001/archpedi.1976.02120130073015

[15] Wayne E , Burrington J . Duodenal obstruction by the superior mesenteric artery in children. Surgery. 1972;72(5):762‐768. 5080598

[16] Massoud W . Laparoscopic management of superior mesenteric artery syndrome. Int Surg. 1995;80:322‐327. 8740677

[17] Richardson W , Surowiec W . Laparoscopic repair of superior mesenteric artery syndrome. Am J Surg. 2001;181:377‐378. 11438278 10.1016/s0002-9610(01)00571-2

[18] Gersin K , Heniford B . Laparoscopic duodenojejunostomy for treatment of superior mesenteric artery syndrome. JSLS. 1998;2:281‐284. 9876755 PMC3015298

[19] Ayloo S , Masrur M , Bianco F , et al. Robotic roux‐en‐Y duodenojejunostomy for superior mesenteric artery syndrome: operative technique. J Laparoendosc Adv Surg Tech A. 2011;21(9):841‐844. 21819217 10.1089/lap.2011.0070

[20] Bütter A , Jayaraman S , Schlachta C . Robotic duodenojejunostomy for superior mesenteric artery syndrome in a teenager. J Robot Surg. 2010;4(4):265‐269. 27627956 10.1007/s11701-010-0215-x

[21] Konstantinidis H , Charisis C , Kottos P . Robotic Strong's procedure for the treatment of superior mesenteric artery syndrome. Description of surgical technique on occasion of the first reported case in the literature. Int J Med Robotics Comput Assist Surg. 2018;14:e1876. doi:10.1002/rcs.1876 29168288

[22] Ha C , Alvear D , Leber D . Duodenal derotation as an effective treatment of superior mesenteric artery syndrome: a thirty‐three year experience. Am Surg. 2008;74(7):644‐653. 18646483

