# Supplementary material for: Robotic modified Strong procedure for superior mesenteric artery syndrome
Source: Clin Case Rep. 2023 Jul 17;11(7):e7651. doi: 10.1002/ccr3.7651 (PMC10350670; doi:10.1002/ccr3.7651)
Supplement: Supplementary file 1 — Video S1 [file CCR3-11-e7651-s001.zip › ccr37651-sup-0002-AppendixS1.docx]

The submission site will not let me upload anything exceeding 97 mb total for all files, and I sent 7 emails trying to figure out a way around it, and this is the best I can come up with. Please let me know if you’re not able to download the video from this link and upload it with the submission and manuscript. Thanks.

<https://drive.google.com/file/d/1HJIqIhBmfGV348ChqKZ37qrUTxfopCgk/view>
